# Changer de paradigmes de communication sur le VIH en Afrique

**DOI:** 10.48327/JNZG-J383

**Published:** 2021-02-18

**Authors:** B. Seytre, B.M. Yoro, M.A. Djedou, O.C. Madiarra, K. Mamey, A. Diabagate, K.B. Kouamé, F. Simaga

**Affiliations:** 1 bnscommunication, 7 rue Ledion, 75014 Paris, France; 2 Université Félix Houphouët Boigny, Abidjan, Côte d’Ivoire; 3 Université Peleforo Gon Coulibaly, Korhogo, Côte d’Ivoire; 4 Alliance, Abidjan, Côte d’Ivoire; 5 PNLS, Abidjan, Côte d’Ivoire; 6 Onusida, Genève, Suisse

**Keywords:** VIH, Sida, Communication, Stratégie, Prévention, Côte d’Ivoire, Afrique, HIV, Aids, Communication, Strategy, Prevention

## Abstract

Nous avons mené en Côte d’Ivoire une étude socio-anthropologique sur la base de laquelle nous proposons une stratégie de communication pour augmenter la demande de tests de dépistage du VIH, notamment chez les jeunes hommes. L’étude montre que l’existence et la fonction du test sont largement connues, que la peur de la mort et de la stigmatisation est le principal obstacle au passage du test, que les hommes sont davantage réticents au test que les femmes et que la peur de la mort liée au sida est particulièrement forte chez les jeunes. La perception des traitements du VIH est contradictoire: une majorité d’enquêtés les connaissent et disent qu’ils permettent de vivre en bonne santé, mais le VIH est toujours fréquemment associé à la mort. Nous préconisons une reprise de la communication de masse sur le VIH, abandonnée au profit de programmes ciblés, afin d’expliquer les traitements, de modifier l’image de l’infection à VIH et d’encourager ainsi toutes les personnes sexuellement actives à passer le test, surtout celles qui ont couru un risque d’infection. Nous préconisons également des messages ciblés en direction des hommes.

## Abstract (en)

We conducted a socio-anthropological study in Ivory Coast to determine recommendations for a communication strategy aimed at increasing HIV testing, especially among young men. The study results showed that the existence of HIV testing is widely known as are the reasons for getting tested. Fear of death and of stigmatization is the main obstacle to testing. Men are more likely than women to be reluctant to undergo HIV testing, and the fear of dying of AIDS is especially strong among young people. Perceptions of HIV treatments are contradictory: while most of those interviewed know about HIV treatments and say that treatment makes it possible to maintain good health, HIV infection is still frequently associated with death. We recommend reinitiating the mass communication campaign around HIV that was abandoned in order to focus on targeted programs, in order to change perceptions of HIV infection and encourage people who are sexually active to get tested, especially those practicing risky behaviors. We also recommend developing communication messages targeting young men.

## Introduction

L’Onusida et le Fonds mondial contre le sida, le paludisme et la tuberculose nous nous ont demandé de mener en Côte d’Ivoire une étude pour proposer une stratégie de communication destinée à augmenter la demande de tests de dépistage du VIH, notamment chez les jeunes hommes, applicable dans le pays et dans toute l’Afrique de l’ouest et du centre.

L’insuffisance de cette demande de tests est le premier obstacle à l’atteinte de l’objectif dit « *90/90/90* » fixé par l’Onusida: 90 % des personnes vivant avec le VIH (PVVIH) connaissent leur statut, 90 % des PVVIH dépistées sont sous traitement antirétroviral et la charge virale est indétectable^1^ chez 90 % de ces dernières. Selon l’Onusida, le pourcentage de PVVIH connaissant leur statut est nettement inférieur en Afrique centrale et occidentale qu’en Afrique orientale et australe. En 2016, il était par exemple de 17 % chez les hommes et 47 % chez les femmes en Sierra Leone, 19 % et 34 % au Congo et 42 % et 46 % en RDC^2^. Ce taux est partout inférieur chez les hommes que chez les femmes. En Côte d’Ivoire, fin 2018 61 % des PVVIH connaissaient leur statut (70 % chez les femmes et 49 % chez les hommes)^3^.

Selon Levin-Zamir et Wills le modèle de santé de toute culture peut être décomposé en quatre segments, la cause des maladies, leurs symptômes, leur diagnostic et leur traitement, et quand la médecine moderne et la médecine traditionnelle se rencontrent « *ce qui est le plus difficile à modifier est la perception de la cause d’une maladie, qui est étroitement associée à l’identité socioculturelle* »^4^. Une condition de la pertinence de la communication sur le test de dépistage du VIH, et donc sur la cause d’une maladie, était qu’elle s’appuie sur une étude de l’identité socioculturelle de la population cible.

C’est la raison pour laquelle, nous inscrivant dans une approche de culture en santé (*health literacy*) définie comme la prise en compte des « *compétences intellectuelles* et *sociales qui déterminent la motivation et la possibilité qu’ont les personnes à obtenir, comprendre et utiliser l’information de façon à favoriser et conserver une bonne santé* », nous avons appuyé notre travail sur les résultats d’une enquête socio-anthropologique^5^.

## MatÉriel et mÉthodes

Une mission préparatoire au cours de laquelle nous avons rencontré environ 150 acteurs de la lutte contre le VIH dans différents villes et villages de Côte d’Ivoire nous a permis de définir les objectifs de l’étude, qui ne porterait pas uniquement sur les perceptions du test de dépistage du VIH, mais également sur celles de l’infection à VIH, du sida et des traitements antirétroviraux contre le VIH (ARV), chez les jeunes hommes et dans l’ensemble de la population.

L’enquête socio-anthropologique a été menée en juin-juillet 2018 par l’Institut des sciences anthropologiques de développement de l’université Houphouët-Boigny (Abidjan), selon un protocole approuvé le 21 juin 2018 par le Comité national d’éthique. Elle a consisté en la tenue de focus groupes (FG) et d’entretiens semi-directifs dans des quartiers populaires et résidentiels des agglomérations d’Abidjan, Bouaké, Korhogo et San Pédro, ainsi que dans un village rattaché à chacune de ces villes. 38 FG ont réuni 304 hommes et femmes séparément, par tranches d’âge: 15-19 ans, 20-34 ans, 35-49 ans et 50 ans et plus. 26 FG ont concerné les deux tranches d’âge les plus jeunes, dont 16 FG hommes et 10 FG femmes. 117 personnes ont été rencontrées en entretiens individuels semi-directifs: 22 agents de santé et membres d’ONG, 20 agents communautaires, 10 tradipraticiens et 65 cas emblématiques ayant participé à des FG.

Les FG et entretiens individuels ont été enregistrés et transcrits, puis ont fait l’objet d’une analyse thématique dont les thèmes étaient les intitulés du guide d’entretien, doublée d’une analyse de contenu prenant en compte toutes les informations.

## RÉsultats

### Perception des tests de dépistage du VIH

Dans leur grande majorité, les enquêtés connaissaient un lieu de dépistage du VIH, confirmant ainsi les résultats de l’enquête MICS 2016 selon laquelle 74,2 % des femmes et 67,2 % des hommes de 15-49 ans connaissent un lieu où passer le test^6^. La majorité des enquêtés savait, en outre, que le test détecte la présence du VIH dans l’organisme, comme l’avait déjà montré une étude en 2000^7^. « *On te fait un prélèvement de sang pour détecter si tu as le virus du sida dans ton organisme. Quand c’est négatif, ça veut dire que tu n’as pas le machin en toi. Par contre, quand c’est positif, ça veut dire que le virus est là* », a par exemple déclaré un jeune homme de Yopougon (Abidjan).

L’enquête a corroboré les propos de l’ensemble des personnels soignants et membres d’ONG rencontrés au cours de la mission préparatoire selon lesquels le principal obstacle au recours au test est la peur d’apprendre un résultat positif. « *Vu ce que la maladie a fait les années passées, les morts que ça a causés, beaucoup de personnes ont peur de se faire dépister. Le fait de savoir que tu portes déjà le virus, te fait penser au point où tu meurs avant la date que Dieu avait prévue pour toi* », selon un jeune homme de Bouaké. « *Si tu es déclaré séropositif, les gens vont s’éloigner de toi et tu risques de vite mourir à cause de ça* », dit un homme de Bouaké. La peur a donc une double composante: peur de la mort, peur de la stigmatisation. Mieux vaut « *ne pas savoir* » est une opinion largement répandue, comme cela avait déjà été relevé par une enquête menée en Côte d’Ivoire en 2017 et qui estimait que « *Je préfère mourir sans savoir si j’ai le sida* » est un propos emblématique^8^.

Selon un agent communautaire de Yopougon, trouver les personnes séropositives de son quartier est de plus en plus difficile: « *Au début ça marchait. Maintenant je peux dire que ça ne marche plus, parce que c’est les mêmes personnes qui se sont fait dépister qui reviennent sur les sites de campagne. On n’arrive pas à trouver de nouvelles personnes* ». Une enquête téléphonique récente en Côte d’Ivoire a mis en évidence que les personnes qui passent le test ne sont pas davantage celles qui ont couru des risques, mais au contraire souvent des gens pas particulièrement exposés^9^. Dans ce même ordre d’idée, les FG de jeunes hommes et femmes ont montré que le fait d’avoir eu des comportements à risque (rapports non protégés, partenaires multiples) incite souvent à ne pas se faire dépister, davantage chez ces jeunes que chez les plus de 35 ans. Une jeune fille de Yopougon a ainsi déclaré: « *Moi j’ai peur, parce que moi je mène ma vie de kpôlé* » (prostituée).

De nombreux enquêtés ont mentionné un manque de confiance dans le respect de la confidentialité par le personnel soignant, ce que l’enquête de 2017 déjà mentionnée avait également souligné.

### Connaissance et représentations du VIH, du sida et des traitements

La quasi-totalité des enquêtés avait entendu parler du sida, comme l’avait déjà montré l’enquête MICS 2016. La majorité des enquêtés fait la distinction entre le VIH, un virus (mot souvent utilisé), et la maladie sida.

Alors que les professionnels de santé rencontrés lors de la mission préparatoire estimaient souvent que la population ne connaissait pas les ARV, l’enquête socio-anthropologique a livré une image différente, marquée par des contradictions.

D’une part, l’existence de traitements proposés par la médecine moderne était largement connue par les enquêtés alors que, d’autre part, l’association du sida à la mort revenait très fréquemment. Ces mots d’un adulte de Korhogo sont représentatifs d’un avis partagé: « *On ne voit aucune possibilité de s’en défaire si ce n’est la mort, car il n’y a pas de possible guérison* ». Un jeune homme du village de Songon dit: « *Le sida est une maladie mortelle, très mortelle d’ailleurs parce que malgré les médicaments qui existent, il continue de faire des ravages.* » Des adolescents de Bouaké ont affirmé que « *avoir le sida, c’est avoir sa mort programmée* », « *si tu as le sida, c’est ta vie qui est finie, tu peux faire quoi encore ?* »

Les enquêtés qui connaissent les ARV savent qu’ils ne guérissent pas la personne infectée, mais qu’ils « *calment* » le VIH selon un mot fréquemment employé et permettent de vivre en bonne santé. Dans toutes les tranches d’âge, ils ont majoritairement affirmé qu’en revanche les médicaments traditionnels ou la prière guérissent.

« *Pour moi, la médecine moderne a échoué sur le côté de la guérison totale du sida. Parce qu’avant le sida, il y a eu le cancer et ils ont trouvé le remède. Mais jusqu’à présent, le sida, ils ont échoué. Donc pour moi, c’est la médecine traditionnelle qui peut soigner ça* » a déclaré un jeune homme de Yopougon. Une femme de Bouaké: « *J’ai entendu parler qu’il y a des gens qui ont été guéris du sida peut-être par la prière. Ils sont repartis faire leur test, on a vu qu’il n’y a plus de sida. D’autres aussi, on a dit que par les tradipraticiens avec leurs médicaments, ils ont été guéris.* » Un homme de San Pédro: « *Moi, je sais qu’il y a des témoignages à l’église.* » Une femme de Korhogo: « *Pour moi, Dieu guérit la maladie. Les tradipraticiens aussi le font, ils guérissent aussi la maladie. Mais au niveau de la médecine moderne, pour moi, ce sont des calmants. Mais je n’ai pas encore entendu qu’au niveau de la médecine, le sida a été totalement guéri.* »

Lors de la mission préparatoire, des personnels de santé ont rapporté des cas de patients qui ont arrêté leurs ARV pour se tourner vers des guérisseurs ou des camps de prière.

### Les perceptions des jeunes

L’association du VIH à la mort était nettement plus forte chez les 15-19 ans que dans les autres tranches d’âge, ce qui confirme les témoignages recueillis pendant la mission préparatoire. Les plus jeunes ignorent fréquemment qu’un PVVIH peut vivre en bonne santé avec les ARV. VIH et sida sont le plus souvent indissociables, celui-ci étant « *une maladie incurable qui n’a pas de médicament* ».

Les souvenirs de ce qu’ils ont appris à l’école se résument à une peur de la maladie et la nécessité de se protéger. « *On nous a dit que c’était un virus dangereux, dangereux, dangereux. Contagieux qui se transmet aussi par le sang. Il faut faire très attention.* » dit un adolescent de San Pédro. « *À l’école primaire, on nous a appris que le sida est une maladie contagieuse. Donc quand quelqu’un a ça, il faut l’éviter.* », selon une adolescente de Cocody (Abidjan). « *Ce qu’on nous a dit à l’école, c’est que le VIH/Sida est une maladie qui contamine tout le monde. C’est une maladie dangereuse.* », se souvient une adolescente de Yopougon (Abidjan).

### Les perceptions et attitudes des hommes

Comme ceux de plusieurs études précédentes, nos résultats montrent que les hommes se considèrent souvent comme moins susceptibles de tomber malades que les femmes, fréquentent moins qu’elles les structures de santé et sont davantage qu’elles réticents à passer le test de dépistage du VIH^10^. Des enquêtés ont apporté des explications biologiques: « *Bon, moi, je me dis que le système immunitaire des hommes est plus résistant que celui des femmes* » a assuré un homme de plus de 50 ans de Bouaké, un habitant du village de Songon, dans la tranche 35-49 ans, précisant « *Les globules blancs de l’homme sont plus forts que pour la femme* ».

De nombreux témoignages ont mis en relief que les hommes recourent davantage que les femmes à la médecine traditionnelle lorsqu’ils sont malades, certains pensant même se protéger contre le VIH par des techniques de « blindage » consistant à se laver avec des mixtures de plantes, nos résultats rejoignant ainsi les conclusions d’une étude précédente^8^.

## Discussion

Les résultats de l’étude socio-anthropologique fournissent des indications essentielles pour la définition d’une stratégie de communication.

La première observation est que la population connaît largement l’existence des tests de dépistage du VIH et leur fonction, la détection du virus du sida. On ne peut donc pas voir dans l’ignorance ou l’incompréhension du test un obstacle à un meilleur dépistage et des messages de communication destinés à faire connaître le test seraient inutiles. Le facteur déterminant du refus de faire un test étant la peur, c’est à celle-ci que la communication doit répondre.

La prise de risque a été évoquée comme une raison de ne pas faire le test, notamment chez les jeunes. Une étude menée en 2007 en Afrique du Sud avait tiré des conclusions similaires en associant le fait de ne jamais avoir passé le test à un âge de moins de 23 ans, des relations sexuelles dans les six derniers mois et la fréquentation de plusieurs partenaires^11^. Des messages de communication reprenant l’idée « tu as couru un risque, fais ton test » seraient donc contre-productifs.

Les ARV sont aujourd’hui largement et gratuitement accessibles en Côte d’Ivoire, même si le pays ne bénéficie pas des molécules les plus récentes. 84 % des PVVIH connaissant leur statut étaient sous ARV fin 2018^12^. Nous ignorons le pourcentage de ces personnes traitées parmi lesquelles la charge virale du VIH était indétectable, ce qui est considéré comme la vérification de l’efficacité du traitement, mais elle l’était chez 77 % des 73 % de personnes sous ARV qui avaient bénéficié d’une mesure de cette charge virale, selon les données du PNLS. La grande majorité des personnes connaissant leur infection par le VIH bénéficie donc aujourd’hui de traitements, efficaces chez la majorité d’entre elles.

Les représentations de l’infection à VIH sont cependant contradictoires puisqu’on constate, d’une part, une connaissance assez répandue de l’existence des ARV et du fait qu’ils permettent de vivre en bonne santé et, d’autre part, la persistance de l’association du VIH au sida et du sida à la mort.

Nous touchons probablement ici la difficulté d’appréhender la notion de chronicité dans la culture de santé africaine^13,14^. Selon Sabrina Fonga: « *On conçoit tout à fait être atteint d’une maladie aiguë par une cause naturelle. En revanche, si celle-ci persiste ou devient chronique, le patient y voit une dimension surnaturelle.* »^15^ Pour Cantrelle et Locoh, « *on retrouve une dichotomie entre maladies ‘naturelles’ et maladies ‘provoquées’ par la sorcellerie, les génies, etc.* »^16^ L’infection à VIH, dont les médecins disent eux-mêmes qu’elle est incurable, tombera dans la catégorie des maladies surnaturelles.

Des traitements contre le sida qui permettent de vivre en bonne santé sans guérir suscitent ainsi l’incompréhension et le doute. Les traitements de la médecine moderne « calment », certes, le virus… mais éloignent-ils la mort ?

La communication ne doit donc pas se contenter de dire qu’il existe des ARV contre le VIH, information déjà largement connue, mais s’attacher à faire accepter la notion d’infection chronique non curable mais pour laquelle il existe des traitements. Les ARV ne permettent pas seulement de vivre en bonne santé, mais aussi de vivre aussi longtemps que tout le monde.

La bonne connaissance du fait que le VIH, un virus, est différent de la maladie sida est un point d’appui pour expliquer que l’infection à VIH ne mène pas inéluctablement au sida: entre le VIH et le sida, il y a les ARV.

Enfin, notre étude confirme que les idées fausses sur l’infection à VIH sont plus prégnantes chez les jeunes que chez les adultes. Une analyse des livres scolaires a montré que ceux-ci présentent toujours l’évolution de l’infection à VIH vers la mort comme inéluctable, ne mentionnent généralement pas les traitements et affirment même parfois qu’il n’en existe pas, ce qui alimente les idées erronées^17^.

## Conclusion

Des facteurs structurels limitent bien sûr la demande du test de dépistage du VIH, dont son accessibilité et les conditions de son passage, mais notre enquête montre que les représentations de l’infection à VIH sont un des principaux obstacles à la progression de la demande de test. Elle souligne aussi que pour atteindre l’objectif de 90 % des PVVIH connaissant leur statut il ne suffira pas d’augmenter la demande globale de tests, mais il faudra amener les personnes qui se sont exposées à l’infection à se faire dépister, celles qui ont le plus de raison de redouter un résultat positif et dont une partie est détournée du test par la peur. L’enjeu de la communication est donc de convaincre que lorsqu’on a couru un risque il est dans son propre intérêt de connaître sa séropositivité.

Cet intérêt est double et répond aux deux types de peurs liées au passage du test. D’une part, un traitement efficace permet de vivre en bonne santé et repousse la mort. D’autre part, un traitement qui amène la charge virale sous le niveau de détectabilité supprime le risque de transmission sexuelle du virus, ce qui est un argument contre la crainte de la stigmatisation^18,19^.

Pendant les deux premières décennies de lutte contre le sida en Afrique, des campagnes de communication de masse ont sensibilisé au fait que c’est une maladie transmissible et mortelle, dont il faut se protéger. Les images de malades cachectiques des panneaux publicitaires de l’époque sont encore dans les mémoires. Puis la communication de masse a quasiment cessé, au bénéfice de programmes de communication de proximité ciblant des populations clés et vulnérables.

Notre étude montre que les représentations qui détournent du test sont partagées par toute la population, hommes et femmes de tous âges, avec une fréquence plus élevée des idées fausses parmi les jeunes et des réticences spécifiques aux hommes. L’augmentation de la demande de test passe aujourd’hui par la reprise d’une communication de masse destinée à transformer ces représentations et qui pourrait être résumée en un slogan: « VIH, bonne nouvelle: il y a des traitements ! »

Nous proposons comme axes de communication :

- Distinguer VIH et sida et bannir les expressions telles que « VIH/sida », « attraper le sida », « protège-toi du sida ». L’évolution vers le sida n’est pas une fatalité.
- Populariser le fait que les ARV permettent de vivre en bonne santé, aussi longtemps que tout le monde. Une infection peut être chronique et contrôlée.
- Associer systématiquement l’avantage de bénéficier d’un traitement aux messages de promotion du test. Une personne inquiète a intérêt à passer le test.
- S’inscrire dans la campagne U=U (undetectable=untransmissible) expliquant qu’un traitement dont l’efficacité est contrôlée supprime les risques de transmission sexuelle du VIH.

Des messages spécifiques seront adressés aux hommes, pour tourner en dérision leurs croyances en l’invulnérabilité.

Enfin, des efforts particuliers doivent être faits en direction des jeunes en utilisant des outils de communication adaptés et en réformant les manuels scolaires.

Ce travail est, à notre connaissance, la seule élaboration d’une stratégie de communication de masse sur le VIH basée sur une enquête socio-anthropologique.

## Conflits D'intérêts

Travail financé par le Fonds mondial contre le sida, le paludisme et la tuberculose.

Les auteurs ne déclarent aucun conflit d’intérêt.
